# Development and validation of a predictive model of abnormal uterine bleeding associated with ovulatory dysfunction: a case-control study

**DOI:** 10.1186/s12905-023-02589-5

**Published:** 2023-10-12

**Authors:** Yue Zhang, Zhou Luo, Yingxian Jia, Yunxiu Zhao, Yizhou Huang, Fei Ruan, Qian Ying, Linjuan Ma, Jie Luo, Jianhong Zhou

**Affiliations:** 1 Women’s Hospital, Zhejiang University School of Medicine, Hangzhou, 310006 Zhejiang China; 2 Zhejiang Provincial Clinical Research Center for Obstetrics and Gynecology, Zhejiang, China; 3 Guizhou Provincial People’s Hospital, Guiyang, 550001 Guizhou China; 4 Zhejiang Cancer Hospital, Hangzhou, 310022 Zhejiang China

**Keywords:** Abnormal uterine bleeding associated with ovulatory dysfunction, Heavy menstrual bleeding, Influencing factors, Predictive model

## Abstract

**Background:**

Abnormal uterine bleeding associated with ovulatory dysfunction (AUB-O) is a typical gynecological disease that can affect women of various ages. Being able to identify women at risk of AUB-O could allow physicians to take timely action. This study aimed to identify the influencing factors of AUB-O in Chinese women, and then develop and validate a predictive model.

**Methods:**

In this multicenter case–control study, 391 women with AUB-O and 838 controls who came from nine hospitals in Zhejiang province were recruited between April 2019 and January 2022. All the participants completed a structured questionnaire including general characteristics, lifestyle and habits, menstrual and reproductive history, and previous diseases. The predictive model was developed on a group of 822 women and validated on a group of 407 women. Logistic regression was adopted to investigate the influencing factors and develop the model, and validation was then performed.

**Results:**

The independent predictive factors of AUB-O were age (OR 1.073, 95% CI 1.046—1.102, *P* < 0.001), body mass index (OR 1.081, 95% CI 1.016—1.151, *P* = 0.015), systolic blood pressure (OR 1.016, 95% CI 1.002—1.029, *P* = 0.023), residence (OR 2.451, 95% CI 1.727—3.478, *P* < 0.001), plant-based diet (OR 2.306, 95% CI 1.415—3.759, *P* < 0.001), fruits eating (OR 1.887, 95% CI 1.282—2.776, *P* = 0.001), daily sleep duration (OR 0.819; 95% CI 0.708—0.946, *P* = 0.007), multiparous (parity = 1, OR 0.424, 95% CI 0.239—0.752, *P* = 0.003; parity > 1, OR 0.450, 95% CI 0.247—0.822, *P* = 0.009), and history of ovarian cyst (OR 1.880, 95% CI 1.305—2.710, *P* < 0.001). The predictive ability (area under the curve) in the development group was 0.77 (95% CI 0.74—0.81), while in the validation group it was 0.73 (95% CI 0.67—0.79). The calibration curve was in high coincidence with the standard curve in the development group, and similar to the validation group. A tool for AUB-O risk calculation was created.

**Conclusions:**

Nine influencing factors and a predictive model were proposed in this study, which could identify women who are at high risk of developing AUB-O. This finding highlights the importance of early screening and the lifelong management of ovulatory disorders for women.

**Supplementary Information:**

The online version contains supplementary material available at 10.1186/s12905-023-02589-5.

## Introduction

Abnormal uterine bleeding (AUB) is a widespread gynecological disease among women all over the world. The prevalence of AUB among women of reproductive age ranges from 20 to 35%, and it is considerably higher in adolescents and perimenopausal women, affecting their life quality and work efficiency to various degrees [1, 2]. Among the classification of causes of AUB (PALM-COEIN, which includes polyp, adenomyosis, leiomyoma, malignancy and hyperplasia, coagulopathy, ovulatory dysfunction, endometrial, iatrogenic, and not yet classified) [3], abnormal uterine bleeding associated with ovulatory dysfunction (AUB-O) is the most common, with a prevalence of approximately 50% in Chinese women [4]. AUB-O is typically characterized by unpredictable timing of menstruation and variable volume, which can sometimes cause heavy menstrual bleeding (HMB) or amenorrhea [5].

Ovulatory dysfunction can result from both physiological and pathological conditions that affect any section of the hypothalamic-pituitary-ovarian axis such as polycystic ovary syndrome (PCOS), premature ovarian failure (POI) and hyperprolactinemia [6]. Most ovulatory disorders are accompanied by AUB symptoms, ranging from occasionally changeable cycle and period to anovulatory [7]. A detailed medical history and physical examination, along with the proper laboratory and imaging studies, will assist with the diagnosis of AUB-O [6].

For a long time, the confusing concept of AUB, as well as challenges in diagnosing ovulatory disorders, hampered the advancement of AUB-O research. There has been relatively little research into the influencing factors for AUB-O. Rezende et al. reported that the prevalence rate of AUB was related to socioeconomic conditions [8]. According to a sub-research of Korea Nurses’ Health Study, irregular menstrual cycles are attached to reproductive, lifestyle, and occupational factors [9]. Increasing age was found to be a risk factor for HMB, which is a distressing manifestation of AUB-O with a prevalence of 30% [10–13]. BMI was discovered to be related to menstrual disorders in a Mendelian randomization study [10], and the same finding was observed in a population-based cross-sectional investigation [14]. However, the risk factors of AUB-O are still unclear, and few studies have been performed to construct a predictive model for AUB-O.

To our best knowledge, this was the first study aimed to investigate the influencing factors and develop a predictive model of AUB-O based on a multicenter case–control study among Chinese women. This study may assist more patients and physicians in identifying AUB-O risk factors and taking appropriate preventative and therapeutic measures to promote female reproductive health while lowering the occurrence of AUB-O and its associated invasive operations.

## Methods

### Design and participants

This multicenter case–control study was initiated by Women’s Hospital, Zhejiang University School of Medicine, and completed collaboratively by 8 other hospitals from across Zhejiang Province. A total of 1789 women who presented to the outpatient clinics of the nine hospitals between April 2019 and January 2022 were recruited. To precisely screen individuals with AUB-O and rule out other causes of AUB, a systematic protocol was used to examine the participants, which comprised of a standardized questionnaire, anthropometric evaluations, physical examination, a transvaginal or transabdominal ultrasound, as well as a battery of laboratory tests such as blood/urine human chorionic gonadotropin, routine blood tests, and blood coagulation indexes. The study was approved by the Human Ethics Committee of Women’s Hospital, Zhejiang University School of Medicine (No. 20180200). All participants provided written informed consent.

The definition of AUB-O is abnormal bleeding from the uterine cavity caused by an ovulatory dysfunction (including anovulation, oligo-ovulation, luteal phase defect, etc.) that is inconsistent with normal menstrual frequency (≥ 24 to ≤ 38 days), regularity (shortest to longest cycle variation: ≤ 7 days), duration (≤ 8 days), and volume (patient determined) [1, 4, 5]. The selection criteria of the AUB-O group were non-pregnant females of reproductive age who lived in Zhejiang Province and suffered from menstrual abnormalities. Women who experienced vaginal bleeding due to other causes of AUB (PALM-CEIN) [3] or caused by other organic diseases such as genital tract trauma were excluded. Controls were chosen from women of reproductive age with normal menstrual cycles, including normal regularity and frequency of menses, duration of flow, and amount of blood loss. Women with any of the following conditions were withdrawn from both the AUB-O and control groups: pregnant, lactating, or postmenopausal; history of malignant tumor; acute and chronic liver diseases; autoimmune diseases; neurological or psychiatric disorders; thyroid diseases; taking hormone therapy or anticoagulants within 3 months. Finally, 391 women with AUB-O and 838 controls were enrolled in this research. To develop the predictive model, the total sample of women was randomly divided into two groups: 2/3 of the study population (development group, *n* = 822) was used for predictive model design, and 1/3 of the study population (validation group, *n* = 407) was used for validation (Supplemental Fig. 1 Flowchart).

### Data collection

The study data was mainly collected by having participants fill out a standardized questionnaire with the assistance of trained medical personnel, comprising sociodemographic information, lifestyle and habits, reproductive history, menstruation, previous diseases, physical examination, assistant examination, and diagnoses and treatment. Body weight, height, and blood pressure were also recorded. BMI was calculated as weight (kg) divided by the square of height (m^2^). The main outcome variable was: the occurrence of AUB-O (Yes/No). From the total set of data, we selected the 49 independent variables listed below for this study:

- General characteristics: age, BMI, systolic blood pressure (SBP), diastolic blood pressure, residence, marriage, education, occupational classification [15], working environment pollutants, and average monthly income.
- Lifestyle and habits: Smoking, alcohol drinking, coffee drinking, tee drinking, diet, fatty food eating, pickled food eating, vegetables eating, fruits eating, physical exercise, dyed hair within 1 year, permed hair within 1 year, used hair gel within 6 months, used nail polish within 6 months, used cosmetics within 6 months, daily sleep duration, sleep quality, experienced mood swings within 6 months.
- Menstrual and reproductive history: age of menarche, dysmenorrhea, sexual experience, age of first pregnancy, age of first birth, gravidity, parity, times of abortion, ectopic pregnancy, lactation, take contraception within 6 months.
- Previous diseases: allergy, ovarian cyst, infertility, hyperprolactinemia, hypertension, diabetes, hyperlipidemia, tuberculosis, asthma, breast diseases.

### Statistical analysis

Statistical analyses were performed using SPSS 27.0 for Windows (IBM, Armonk, NY, USA). Kolmogorov–Smirnov tests were used to determine the normality of the distributions. Data for continuous variables are presented as mean ± SD for normally distributed variables and median (25th percentile 75th percentile) for non-normally distributed variables. Continuous variable comparisons between two groups were evaluated using the t-test for equal variances or the Mann–Whitney U test for unequal variances. Categorical variables were presented as n (%) and compared using Pearson chi-square tests. All variables contained a maximum of 2.77% missing values. For all analyses, a *P*-value of < 0.05 was considered statistically significant.

In the development group, a univariate analysis was adopted to compare the 49 potential influencing factors of AUB-O between the two groups. Variables with *P*-values < 0.20 were included in the multivariate binary logistic regression model (forward LR, PIN = 0.05, POUT = 0.10) to identify predictors of AUB-O. A univariate analysis was then used to compare the homogeneity of the final predictors between the development and validation groups. The receiver operating characteristic curves (ROC) were designed to test the discrimination of the model in the development and validation groups. Area under curves (AUC) were calculated and assessed according to the Swet’s criteria [16], whose values range from 0.5–0.6 (bad), 0.6–0.7 (poor), 0.7–0.8 (satisfactory), 0.8–0.9 (good), and 0.9–1.0 (excellent). The maximum Youden index, the optimal cutoff value, sensitivity and specificity were calculated. The Hosmer–Lemeshow Goodness-of-Fit Test and calibration curves were performed to assess the consistency of the predicted risk and actual risk, with a *P*-value > 0.05 indicating satisfactory discrimination. Finally, a tool for AUB-O risk calculation was designed by Excel for the purpose of clinical use.

## Results

### Univariate analysis of potential influencing factors associated with AUB-O

A total of 1229 women participated in the study, including 822 women in the development group and 407 women in the validation group. Women with AUB-O accounted for 32.00% of the development group and 31.45% of the validation group. In the development group, the average ages of the AUB-O group and control group were 41.31 ± 9.50 years and 35.95 ± 8.17 years (*P* < 0.001), and the median hemoglobin of the two groups was 115 (90 131) g/L and 128 (119 135) g/L (*P* < 0.001) (Supplemental Table 1).

The results of the univariate analysis were displayed in Supplemental Table 1. We identified 27 variables (*P* < 0.20) potentially associated with AUB-O among 49 variables to develop the predictive model by logistic regression analysis: age, BMI, SBP, residence, education, occupational classification, working environment pollutants, average monthly income, smoking, alcohol drinking, coffee drinking, tee drinking, diet, fruits eating, permed hair within 1 year, used hair gel within 6 months, used nail polish within 6 months, used cosmetics within 6 months, daily sleep duration, sleep quality, experienced mood swings within 6 months, age of menarche, parity, lactation, history of ovarian cyst, hypertension, history of breast diseases.

### The influencing factors and final predictive model of AUB-O

As shown in Table 1, nine variables were found to be the independent influencing factors of AUB-O in the final predictive model: age, BMI, SBP, residence, diet, fruits eating, daily sleep duration, parity and history of ovarian cyst. There was no significant difference in any of the nine predictors between the development and validation groups (Table 2).

**Table 1:** Risk predictive model for AUB-O

Predictor	β	OR (95%CI)	*P*-value
**Age**	0.071	1.073 (1.046—1.102)	** < 0.001**
**BMI**	0.078	1.081 (1.016—1.151)	**0.015**
**SBP**	0.016	1.016 (1.002—1.029)	**0.023**
**Residence**			
Urban		1 (Ref)	
Rural	0.896	2.451 (1.727—3.478)	** < 0.001**
**Diet**			**0.003**
With meat and vegetables		1 (Ref)	
Meat-based diet	-0.002	0.998 (0.495—2.010)	0.995
Plant-based diet	0.836	2.306 (1.415—3.759)	** < 0.001**
**Fruits eating**			
≥ 4 times per week		1 (Ref)	
** < **4 times per week	0.635	1.887 (1.282—2.776)	**0.001**
**Daily sleep duration**	-0.200	0.819 (0.708—0.946)	**0.007**
**Parity**			**0.012**
0		1 (Ref)	
1	-0.858	0.424 (0.239—0.752)	**0.003**
> 1	-0.798	0.450 (0.247—0.822)	**0.009**
**History of ovarian cyst**			
No		1 (Ref)	
Yes	0.631	1.880 (1.305—2.710)	** < 0.001**

*Abbreviations*: *BMI* body mass index, *SBP* systolic blood pressure

**Table 2:** Comparison of predictors between development and validation groups

Predictor	Development Group (*n* = 822)	Validation Group (*n* = 407)	*P*-value
**Age (year) **^**a**^	37.67 ± 8.97	37.67 ± 9.29	0.994
**BMI (kg / m**^**2**^**) **^**a**^	21.98 ± 2.85	21.88 ± 2.99	0.570
**SBP (mmHg) **^**b**^	116 (108 124)	116(108 126)	0.944
**Residence **^**c**^			0.586
Urban	564 (68.61)	273 (67.08)	
Rural	258 (31.39)	134 (32.92)	
**Diet **^**c**^			0.325
With meat and vegetables	674 (82.00)	321 (78.87)	
Meat-based diet	50 (6.08)	33 (8.11)	
Plant-based diet	98 (11.92)	53 (13.02)	
**Fruits eating **^**c**^			0.989
≥ 4 times per week	646 (78.59)	320 (78.62)	
** < **4 times per week	176 (21.41)	87 (21.38)	
**Daily sleep duration (hour)**^**b**^	8.00 (7.00 8.00)	7.50 (7.00 8.00)	0.379
**Parity **^**c**^			0.697
0	170 (20.68)	90 (22.11)	
1	375 (45.62)	189 (46.44)	
> 1	277 (33.70)	128 (31.45)	
**History of ovarian cyst **^**c**^			0.543
No	613 (74.57)	310 (76.17)	
Yes	209 (25.43)	97 (23.83)	

*Abbreviations*: *BMI* body mass index, *SBP* systolic blood pressure

^a^Mean ± SD; t test

^b^Median (P25 P75); Mann–Whitney U test

^c^n (%); Pearson chi-square test

Logistic regression analysis found a higher risk of AUB-O in women with older age (OR 1.073, 95% CI 1.046—1.102), a higher BMI (OR 1.081, 95% CI 1.016—1.151) and SBP (OR 1.016, 95% CI 1.002—1.029), living in rural areas (OR 2.451, 95% CI 1.727—3.478), having a plant-based diet (OR 2.306, 95% CI 1.415—3.759), eating fruits < 4 times per week (OR 1.887, 95% CI 1.282—2.776) and having a history of ovarian history (OR 1.880, 95% CI 1.305—2.710). On the contrary, women who were multiparous (parity = 1, OR 0.424, 95% CI 0.239 – 0.752; parity > 1, OR 0.450, 95% CI 0.247 – 0.822) and had a longer daily sleep duration (OR 0.819; 95% CI 0.708 – 0.946) were at a decreased risk of AUB-O (Table 1).

The maximum Youden index of the model is 0.42, and the optimal cutoff value is 30.15%, with a sensitivity valued at 0.72 and a specificity valued at 0.71. A tool for AUB-O risk calculation was shown in Additional file 2.

### Validation of the predictive model of AUB-O: discrimination and calibration

**Fig. 1:**
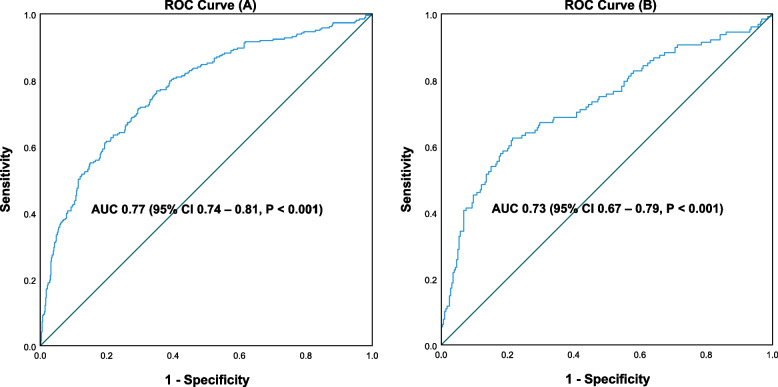
ROC curve of the predictive model for the development group (**A**) and the validation group (**B**)

In the development group, the AUC of ROC was 0.77 (95% CI 0.74 – 0.81), indicating satisfactory discrimination and predictive ability of the model. The AUC of ROC in the validation group obtained 0.73 (95% CI 0.67 – 0.79), which was also satisfactory (Fig. 1).

**Fig. 2:**
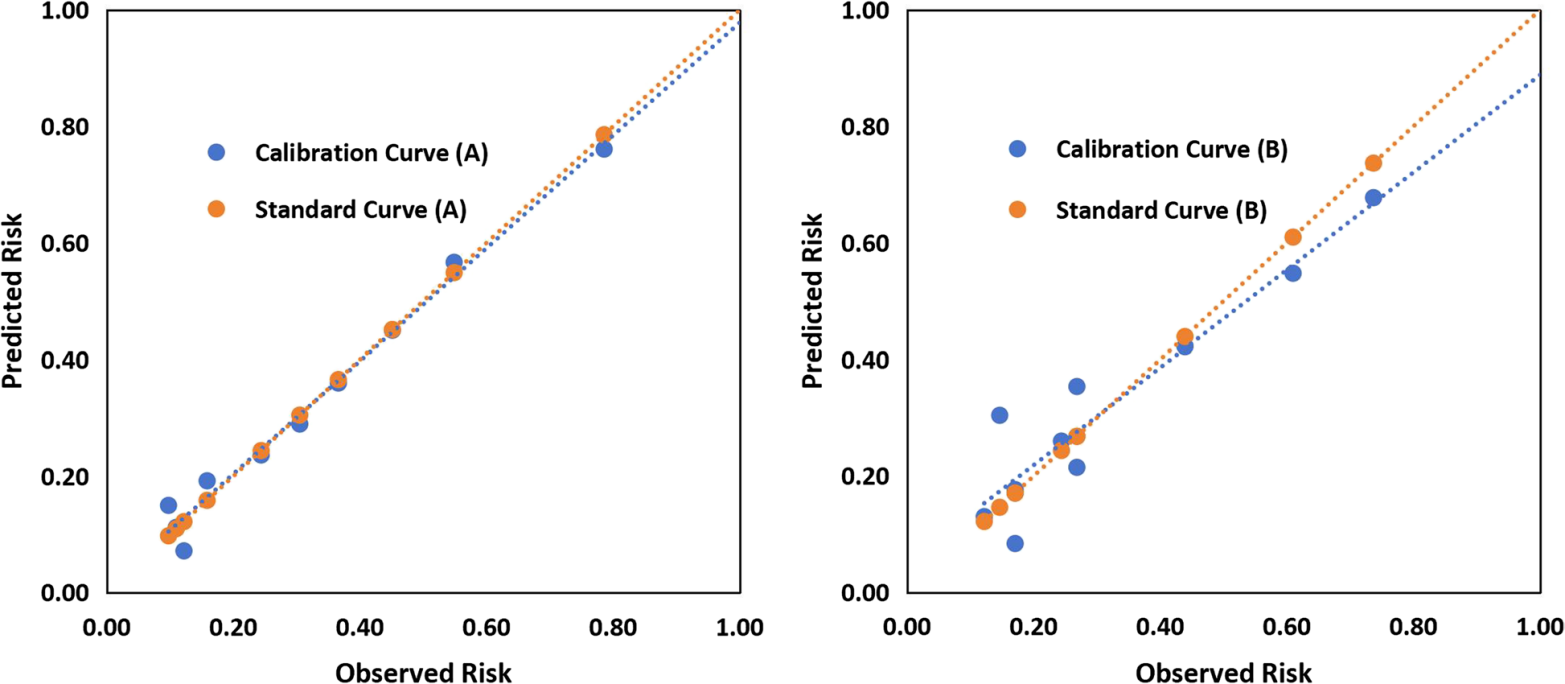
Calibration curve of the predictive model for the development group (**A**) and the validation group (**B**)

The calibration curve was in high coincidence with the standard curve in the development group, suggesting the predicted risk of the model is highly consistent with the actual AUB-O risk. Meanwhile, the calibration curve and the standard curve were close to each other in the validation group (Fig. 2).

## Discussion

A predictive model was developed based on our case–control study, with satisfactory predictive ability and calibration. The predictors involved in the model are age, BMI, SBP, residence, diet, fruits eating, daily sleep duration, parity, and history of ovarian cyst.

Due to the imprecise terminologies and definitions and the lack of a standardized etiologic classification system in the past, the investigation and management of AUB were hampered for a long time [1–4, 8]. To our knowledge, there are only a few studies focused on the prediction of AUB and no predictive model for AUB-O. Xu et al. proposed the combination of vaginal ultrasonography and bleeding pattern had a good predictive value for AUB. They discovered that BMI, dysmenorrhea, endometrial thickness, diabetes, hypertension, and polycystic ovarian syndrome were related factors of AUB [17]. An analysis of medication-induced heavy bleeding in women with severe mental illnesses conducted in China revealed that some metabolic profiles and antipsychotic therapies were risk factors for heavy bleeding [18]. Four cross-sectional studies [9, 10, 19, 20] conducted in different countries investigated the local prevalence of AUB and associated factors, but with controversial conclusions, suggesting racial differences in AUB prevalence and risk factors might exist.

In our findings, older age was a risk factor of AUB-O. Kazemijaliseh et al. investigated 1393 Iranian women aged 15–45 years to explore the prevalence of AUB and its associated factors, and they observed an increasing prevalence of AUB in older women [10], which agreed with our findings. The Israel researchers carried out an online national questionnaire survey to evaluate menstrual disorders among COVID-19 vaccinated and infected women, and they identified increasing age might be a contributor of AUB after COVID-19 vaccination [13]. However, a Chinese study discovered an inverse relationship between age and HMB in 2356 women aged 18–50 years [20]. The consensus seems to be that ovulatory disorders are more prevalent in females during adolescence and the menopausal transition [7], while more research is still needed to determine the exact mechanisms underlying how menstruation changes as people age.

BMI was another risk factor of AUB-O in our research. Besides aging, the Iranian research also found higher BMI was related to AUB [10]. A Mendelian randomization study [14] investigated 257,193 women of European ancestry in UK Biobank and publicly available genome-wide association studies (GWASs) and revealed that numerous female reproductive disorders are correlated with obesity. Higher BMI was observationally associated with HMB in their study, and leptin and insulin resistance were potential mediators between obesity and female reproductive health [14]. Mena et al. conducted a prospective cohort study based on the Australian Longitudinal Study of Women’s Health (ALSWH) and found that overweight and obese women had a higher risk of irregular periods and HMB, but this effect could be weakened by high levels of physical activity [21]. As estrogen is converted from androstenedione in adipose tissue by aromatase, obese individuals typically have high estrogen levels. In addition, obese women's sex hormone-binding globulin (SHBG) dropped while their insulin levels rose, promoting the production of androgens. Ovulation and menstrual disorders, including irregular bleeding, oligomenorrhoea, and amenorrhea, are caused by these changes in gonadal steroid concentrations connected to obesity [22].

In Korean female adolescents, sleep duration and irregular menstrual cycles were found to be significantly inversely correlated [23], which was in coincidence with our findings. Hall et al. recruited 11 healthy females to receive a 40-h simultaneous polysomnographic sleep monitoring and luteinizing hormone (LH) measurement, women in the early follicular phase experienced a nocturnal decrease in mean LH and LH pulse frequency due to the inhibited LH and gonadotropin-releasing hormone secretion by sleep [24]. Women's reproductive function such as folliculogenesis, ovulation, menstruation, hormone synthesis, and secretion can be hampered by sleep deprivation, disruption, dysrhythmia, and disorders, and the complex molecular-genetic and hormonal pathways play a major role in mediating these relationships [25].

Multiparity was found to be a beneficial factor for AUB-O in our study. He et al. reported a lower chance of gravidity and parity in women with oligomenorrhea [26]. Grimes et al. examined anti-mullerian hormone levels in a subset of premenopausal women in the Nurses’ Health Study II. They discovered positive correlations between parity and both the level of anti-mullerian hormone and the timing of menopause, but these relations vanished after further parity adjustment [27]. Meanwhile, a cross-sectional study found a significant correlation between parity and higher levels of ovarian reserve markers [28]. A population-based cohort study included premenopausal participants aged 25 to 42 years at baseline in the Nurses' Health Study II cohort proposed that parity had an inverse relationship with the risk of early menopause [29]. Similar outcomes were observed in a pooled study carried out by Mishra et al. [30]. Pregnancy inhibits ovulation and may slow the loss of ovarian follicles, which delays menopause [29]. In summary, we speculate that multiparity may have beneficial effects on ovarian function.

Balanced diet is a critical component for the normal functioning of the hypothalamic-ovarian axis, which has considerable promise for improving many chronic gynecologic illnesses and reproductive health [31, 32]. A diet rich in fruits and vegetables has a protective impact that lowers the risk of uterine fibroids and endometriosis [33, 34]. The Mediterranean diet and Dietary Approaches to Stop Hypertension (DASH) diet are two internationally recognized healthy dietary patterns. They have auxiliary therapeutic effects on weight control, prevention and control of cardiovascular diseases, diabetes and many other diseases. Recently, a study reported that these two healthy diets also has the ability to promote the ovarian morphology and function [35]. Compared to the age- and BMI-matched healthy non-vegetarians, women with PCOS who followed an Indian vegetarian diet had greater levels of pro-inflammatory and lower levels of anti-inflammatory markers [36].

Rural Chinese women appeared to be at a higher risk of developing AUB-O than urban residents. There are currently few research focusing on the differences in female reproductive health between urban and rural locations. Rural women, according to Wang et al., have a higher proportion of osteoporosis than women lived in urban areas [37]. A study on Chinese all-cause mortality rate found that the health status of rural residents in China is generally worse than that of urban and suburban residents [38]. In general, the majority of rural Chinese women have lower income and education levels, and poorer health perceptions and medical resources than urban women. The lifestyle disparities between urban and rural women are significant, which may result in physical variations [37].

Despite of the insignificant difference in hypertension between the AUB-O and control groups (Supplemental Table 1), we identified that SBP was a risk factor for AUB-O. In contrast to controls, women with PCOS had significantly higher blood pressure [39]; however, this difference was not seen in women with POI [40]. Estrogen plays a protective effect on the cardiovascular system, ovarian dysfunction could probably interfere with its secretion. Generally, benign ovarian cysts have little effect on ovarian function. However, we found that a history of ovarian cyst was another risk factor of AUB-O. We did not perform any additional pathological classification of ovarian cysts in our study, some of which may impair ovarian ovulation and endocrine function. In the recent Delphi consensus process [7], there was now broad agreement that ovarian tumors, both benign and malignant, may play a role in ovulatory disorders. These findings may prompt more people to consider the impact of an ovarian cyst on ovarian function.

Our study has some limitations. Firstly, recall bias might exist in the data collection process. However, participants completed the questionnaires with the help of trained medical personnel, ensuring the reliability of data. Secondly, limited by the retrospective observational method, our study can only illustrate the association between exposure factors and AUB-O, but cannot prove a causation between them. In addition, the data used to develop and validate the model was completely from China, which might limit the generalizability of the model in other countries. Further research is required in a broader range of ethnicities. Regarding the strength of the study, our predictive model presented a satisfactory prediction and calibration capability with a large sample. Our study proposes the prediction model of AUB-O for the first time, which provides a new tool for public-health workers to assess the risk of AUB-O.

## Conclusion

A predictive model for AUB-O with moderate diagnostic accuracy has been developed. This simple and straightforward tool can assist medical personnel in identifying high-risk groups and taking interventions whenever possible. However, further research is needed to verify and expand our model in the future.

### Supplementary Information

**Additional file 1: Supplemental Fig. 1.** Flowchart. **Supplemental Table 1.** Comparison of potential influencing factors associated with AUB-O between the two groups. **Supplemental Table 2.** Assignment of predictive factors of AUB-O.

**Additional file 2.**

## Data Availability

The datasets used and/or analysed during the current study are available from the corresponding author on reasonable request.

## Abbreviations

AUB: Abnormal uterine bleeding
AUB-O: Abnormal uterine bleeding associated with ovulatory dysfunction
HMB: Heavy menstrual bleeding
PCOS: Polycystic ovary syndrome
POI: Premature ovarian failure
BMI: Body mass index
SBP: Systolic blood pressure
ROC: The receiver operating characteristic curve
AUC: Area under curves
LH: Luteinizing hormone
