# Supplementary material for: Development and validation of a predictive model of abnormal uterine bleeding associated with ovulatory dysfunction: a case-control study
Source: BMC Womens Health. 2023 Oct 12;23:536. doi: 10.1186/s12905-023-02589-5 (PMC10571233; doi:10.1186/s12905-023-02589-5)
Supplement: Supplementary file 1 — Additional file 1: Supplemental Fig. 1. Flowchart. Supplemental Table 1. Comparison of potential influencing factors associated with AUB-O between the two groups. Supplemental Table 2. Assignment of predictive factors of AUB-O. [file 12905_2023_2589_MOESM1_ESM.docx]

**Supplemental Fig. 1** Flowchart


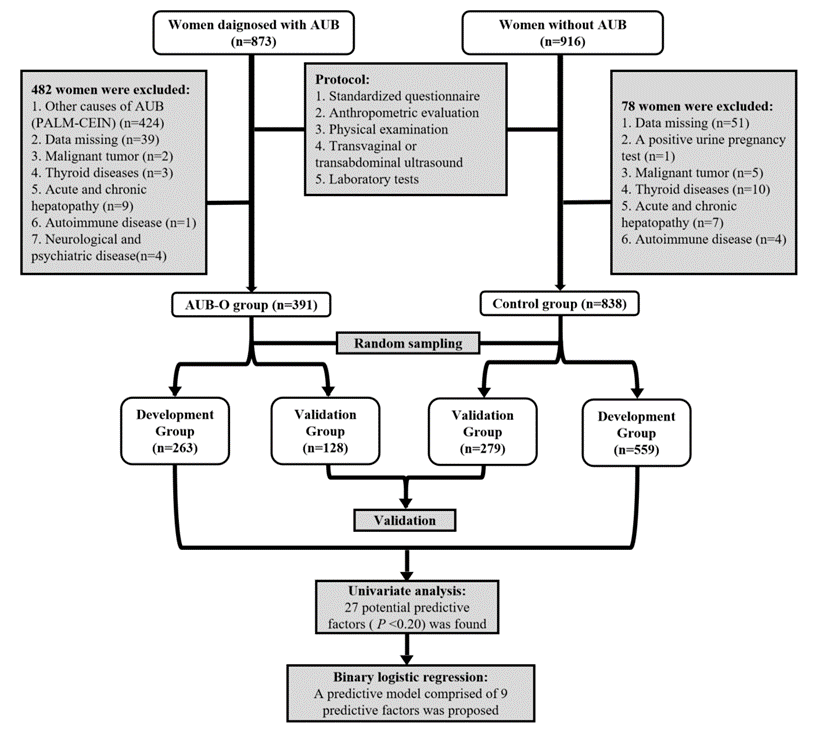


**Supplemental Table 1** Comparison of potential influencing factors associated with AUB-O between the two groups

| **Factor** | **AUB-O**  **(n = 263)** | **Controls**  **(n = 559)** | **p-value** |
| --- | --- | --- | --- |
| **General characteristics** | | | |
| **Age (year)** | 41.31 ± 9.50 | 35.95 ± 8.17 | **< 0.001** |
| **Age (year)** |  |  |  |
| < 18 | 6 (2.28) | 1 (0.18) |  |
| 18-45 | 140 (53.23) | 476 (85.15) |  |
| > 45 | 117 (44.49) | 82 (14.67) |  |
| **BMI (kg / m^2^)** | 22.86 ± 3.09 | 21.56 ± 2.64 | **< 0.001** |
| **Systolic blood pressure (mmHg)** | 120 (110 130) | 113 (107 121) | **< 0.001** |
| **Diastolic blood pressure (mmHg)** | 72 (66 78) | 72 (67 80) | 0.213 |
| **Residence** |  |  | **< 0.001** |
| Urban | 145 (55.13) | 419 (74.96) |  |
| Rural | 118 (44.87) | 140 (25.04) |  |
| **Marriage** |  |  | 0.590 |
| Married | 238 (90.49) | 499 (89.27) |  |
| Unmarried | 25 (9.51) | 60 (10.73) |  |
| **Education** |  |  | **< 0.001** |
| Illiteracy | 15 (5.70) | 8 (1.43) |  |
| Primary school | 47 (17.87) | 49 (8.77) |  |
| Junior high school | 88 (33.46) | 143 (25.58) |  |
| Senior high school | 41 (15.59) | 85 (15.21) |  |
| Junior college | 28 (10.65) | 115 (20.57) |  |
| Bachelor's degree or above | 44 (16.73) | 159 (28.44) |  |
| **Occupational Classification ^a^** |  |  | **< 0.001** |
| Category I | 5 (1.90) | 9 (1.61) |  |
| Category II | 41 (15.59) | 168 (30.05) |  |
| Category III | 4 (1.52) | 11 (1.97) |  |
| Category IV | 78 (29.66) | 156 (27.91) |  |
| Category V | 13 (4.94) | 12 (2.15) |  |
| Category VI | 53 (20.15) | 80 (14.31) |  |
| Category VIII | 69 (26.24) | 123 (22.00) |  |
| **Working environment pollutants** |  |  | 0.085 |
| Yes | 17 (6.46) | 21 (3.76) |  |
| No | 246 (93.54) | 538 (96.24) |  |
| **Average monthly income (RMB ^b^)** |  |  | **< 0.001** |
| < 2000 | 58 (22.05) | 81 (14.49) |  |
| 2000~5000 | 109 (41.44) | 170 (30.41) |  |
| ~10000 | 60 (22.81) | 227 (40.61) |  |
| 10000~20000 | 24 (9.13) | 62 (11.09) |  |
| > 20000 | 12 (4.56) | 19 (3.40) |  |
| **Lifestyle and habits** | | | |
| **Smoking** |  |  | 0.065 |
| Yes | 1 (0.38) | 14 (2.50) |  |
| No | 262 (99.62) | 545 (97.50) |  |
| **Alcohol drinking** |  |  | 0.198 |
| Yes | 15 (5.70) | 46 (8.23) |  |
| No | 248 (94.30) | 513 (91.77) |  |
| **Coffee drinking** |  |  | **< 0.001** |
| Never | 232 (88.21) | 439 (78.53) |  |
| Ever | 10 (3.80) | 64 (11.45) |  |
| Present | 21 (7.98) | 56 (10.02) |  |
| **Tee drinking** |  |  | 0.130 |
| Never | 198 (75.29) | 403 (72.09) |  |
| Ever | 23 (8.75) | 76 (13.60) |  |
| Present | 42 (15.97) | 80 (14.31) |  |
| **Diet** |  |  | **< 0.001** |
| With meat and vegetables | 190 (72.24) | 484 (86.58) |  |
| Meat-based diet | 16 (6.08) | 34 (6.08) |  |
| Plant-based diet | 57 (21.67) | 41 (7.33) |  |
| **Fatty food eating** |  |  | 0.342 |
| ≤ 1 time per week | 231 (87.83) | 470 (84.08) |  |
| 2~3 times per week | 26 (9.89) | 75 (13.42) |  |
| ≥ 4 times per week | 6 (2.28) | 14 (2.50) |  |
| **Pickled food eating** |  |  | 0.471 |
| ≤ 1 time per week | 241 (91.63) | 505 (90.34) |  |
| 2~3 times per week | 20 (7.60) | 44 (7.87) |  |
| ≥ 4 times per week | 2 (0.76) | 10 (1.79) |  |
| **Vegetables eating** |  |  | 0.589 |
| ≤ 1 time per week | 3 (1.14) | 8 (1.43) |  |
| 2~3 times per week | 24 (9.13) | 40 (7.16) |  |
| ≥ 4 times per week | 239 (89.73) | 511 (91.41) |  |
| **Fruits eating** |  |  | **< 0.001** |
| **<** 4 times per week | 82 (31.18) | 94 (16.82) |  |
| ≥ 4 times per week | 181 (68.82) | 465 (83.18) |  |
| **Physical exercise** |  |  | 0.842 |
| Hardly | 108 (41.06) | 211 (37.75) |  |
| Low intensity | 75 (28.52) | 169 (30.23) |  |
| Middle intensity | 67 (25.48) | 150 (26.83) |  |
| High intensity | 13 (4.94) | 29 (5.19) |  |
| **Dyed hair within 1 year** |  |  | 0.855 |
| Yes | 121 (46.01) | 261 (46.69) |  |
| No | 142 (53.99) | 298 (53.31) |  |
| **Permed hair within 1 year** |  |  | **0.016** |
| Yes | 87 (33.08) | 140 (25.04) |  |
| No | 176 (66.92) | 419 (74.96) |  |
| **Used hair gel within 6 months** |  |  | 0.133 |
| Often | 3 (1.14) | 4 (0.72) |  |
| Occasionally | 7 (2.66) | 31 (5.55) |  |
| Never | 253 (96.20) | 524 (93.74) |  |
| **Used nail polish within 6 months** |  |  | **0.041** |
| Often | 9 (3.42) | 14 (2.50) |  |
| Occasionally | 24 (9.13) | 86 (15.38) |  |
| Never | 230 (87.45) | 459 (82.11) |  |
| **Used cosmetics within 6 months** |  |  | **< 0.001** |
| Often | 28 (10.65) | 95 (16.99) |  |
| Occasionally | 32 (12.17) | 119 (21.29) |  |
| Never | 203 (77.19) | 345 (61.72) |  |
| **Daily sleep duration (hours)** | 7.00 (6.50 8.00) | 8.00 (7.00 8.00) | **< 0.001** |
| **Sleep quality** |  |  | **< 0.001** |
| Good or average | 230 (87.45) | 534 (95.53) |  |
| Poor | 33 (12.55) | 25 (4.47) |  |
| **Experienced mood swings within 6 months** |  |  | 0.120 |
| Yes | 34 (12.93) | 96 (17.17) |  |
| No | 229 (87.07) | 463 (82.83) |  |
| **Menstrual and reproductive history** | | | |
| **Age of menarche (year)** | 14.27 ± 1.52 | 13.87 ± 1.32 | **< 0.001** |
| **Dysmenorrhea** |  |  | 0.303 |
| Yes | 23 (8.75) | 62 (11.09) |  |
| No | 240 (91.25) | 497 (88.91) |  |
| **Sexual experience** |  |  | 0.309 |
| Ever | 242 (92.02) | 525 (93.92) |  |
| Never | 21 (7.98) | 34 (6.08) |  |
| **Age of first pregnancy (year)** | 23.69 ± 2.81 | 23.76 ± 3.11 | 0.773 |
| **Age of first birth (year)** | 24.74 ± 2.96 | 24.83 ± 3.09 | 0.726 |
| **Gravidity** |  |  | 0.364 |
| 0 | 36 (13.69) | 97 (17.35) |  |
| 1~3 | 165 (62.74) | 344 (61.54) |  |
| ≥ 4 | 62 (23.57) | 118 (21.11) |  |
| **Parity** |  |  | **0.017** |
| 0 | 42 (15.97) | 128 (22.90) |  |
| 1 | 117 (44.49) | 258 (46.15) |  |
| ≥ 2 | 104 (39.54) | 173 (30.95) |  |
| **Times of abortion** |  |  | 0.383 |
| 0 | 103 (39.16) | 224 (40.07) |  |
| 1 | 84 (31.94) | 154 (27.55) |  |
| ≥ 2 | 76 (28.90) | 181 (32.38) |  |
| **Ectopic pregnancy** |  |  | 0.674 |
| Ever | 12 (4.56) | 22 (3.94) |  |
| Never | 251 (95.44) | 537 (96.06) |  |
| **Lactation** |  |  | **0.014** |
| Ever | 212 (80.61) | 406 (72.63) |  |
| Never | 51 (19.39) | 153 (27.37) |  |
| **Take contraception within 6 months** |  |  | 0.231 |
| Yes | 174 (66.16) | 393 (70.30) |  |
| No | 89 (33.84) | 166 (29.70) |  |
| **Previous disease** | | | |
| **Allergy** |  |  | 0.309 |
| Yes | 21 (7.98) | 34 (6.08) |  |
| No | 242 (92.02) | 525 (93.92) |  |
| **Ovarian cyst** |  |  | **< 0.001** |
| Yes | 96 (36.50) | 113 (20.21) |  |
| No | 167 (63.50) | 446 (79.79) |  |
| **Infertility** |  |  | 0.685 |
| Yes | 2 (0.76) | 10 (1.79) |  |
| No | 261 (99.24) | 549 (98.21) |  |
| **Hyperprolactinemia** |  |  | 1.000 |
| Yes | 0 (0.00) | 1 (0.18) |  |
| No | 263 (100.00) | 558 (99.82) |  |
| **Hypertension** |  |  | 0.063 |
| Yes | 17 (6.46) | 20 (3.58) |  |
| No | 246 (93.54) | 539 (96.42) |  |
| **Diabetes** |  |  | 0.391 |
| Yes | 3 (1.14) | 3 (0.54) |  |
| No | 260 (98.86) | 556 (99.46) |  |
| **Hyperlipidemia** |  |  | 0.597 |
| Yes | 2 (0.76) | 2 (0.36) |  |
| No | 261 (99.24) | 557 (99.64) |  |
| **Tuberculosis** |  |  | 1.000 |
| Yes | 0 (0.00) | 2 (0.36) |  |
| No | 263 (100.00) | 557 (99.64) |  |
| **Asthma** |  |  | 0.334 |
| Yes | 3 (1.14) | 2 (0.36) |  |
| No | 260 (98.86) | 557 (99.64) |  |
| **Breast diseases** |  |  | **0.046** |
| Yes | 28 (10.65) | 37 (6.62) |  |
| No | 235 (89.35) | 522 (93.38) |  |

^a^ According to the Occupational Classification Code of the People's Republic of China (2015 version). Category I: leaders of state organs, party and mass organizations, enterprises, and public institutions; Category II: Professional and technical personnel; Category III: Office staff and related personnel; Category IV: Business and service personnel; Category V: Production personnel in agriculture, forestry, animal husbandry, fishery, and water conservancy; Category VI: Production and transportation equipment operators; Category VII: Military; Category VIII: Occupations not convenient to be categorized.

^b^ RMB: Ren Min Bi, the currency of the People’s Republic of China.

**Supplemental Table 2** Assignment of predictive factors of AUB-O

| **Predictor** | **Variable** | **Assignment and unit** |
| --- | --- | --- |
| **Age** | X_1_ | year |
| **BMI** | X_2_ | kg / m^2^ |
| **Systolic blood pressure** | X_3_ | mmHg |
| **Residence** | X_4_ | Urban = 0, rural = 1 |
| **Diet** | X_5_ | with meat and vegetables = 1, meat-based diet = 2, plant-based diet = 3 |
| **Fruits eating** | X_6_ | ≥ 4 times per week = 0, < 4 times per week = 1 |
| **Daily sleep duration** | X_7_ | hour |
| **Parity** | X_8_ | 0 = 1, 1= 2, ≥ 1 = 3 |
| **History of ovarian cyst** | X_9_ | no = 0, yes = 1 |
| **AUB-O** | Y | no = 0, yes = 1 |
